# Syndecan-1 Enhances Proliferation, Migration and Metastasis of HT-1080 Cells in Cooperation with Syndecan-2

**DOI:** 10.1371/journal.pone.0039474

**Published:** 2012-06-26

**Authors:** Bálint Péterfia, Tibor Füle, Kornélia Baghy, Krisztina Szabadkai, Alexandra Fullár, Katalin Dobos, Fang Zong, Katalin Dobra, Péter Hollósi, András Jeney, Sándor Paku, Ilona Kovalszky

**Affiliations:** 1 1st Department of Pathology and Experimental Cancer Research, Semmelweis University, Budapest, Hungary; 2 Division of Pathology, Department of Laboratory Medicine, Karolinska Institutet, Huddinge, Stockholm, Sweden; University of Hong Kong, Hong Kong

## Abstract

Syndecans are transmembrane heparan sulphate proteoglycans. Their role in the development of the malignant phenotype is ambiguous and depends upon the particular type of cancer. Nevertheless, syndecans are promising targets in cancer therapy, and it is important to elucidate the mechanisms controlling their various cellular effects. According to earlier studies, both syndecan-1 and syndecan-2 promote malignancy of HT-1080 human fibrosarcoma cells, by increasing the proliferation rate and the metastatic potential and migratory ability, respectively. To better understand their tumour promoter role in this cell line, syndecan expression levels were modulated in HT-1080 cells and the growth rate, chemotaxis and invasion capacity were studied. For in vivo testing, syndecan-1 overexpressing cells were also inoculated into mice. Overexpression of full length or truncated syndecan-1 lacking the entire ectodomain but containing the four juxtamembrane amino acids promoted proliferation and chemotaxis. These effects were accompanied by a marked increase in syndecan-2 protein expression. The pro-migratory and pro-proliferative effects of truncated syndecan-1 were not observable when syndecan-2 was silenced. Antisense silencing of syndecan-2, but not that of syndecan-1, inhibited cell migration. In vivo, both full length and truncated syndecan-1 increased tumour growth and metastatic rate. Based on our in vitro results, we conclude that the tumour promoter role of syndecan-1 observed in HT-1080 cells is independent of its ectodomain; however, in vivo the presence of the ectodomain further increases tumour proliferation. The enhanced migratory ability induced by syndecan-1 overexpression is mediated by syndecan-2. Overexpression of syndecan-1 also leads to activation of IGF1R and increased expression of Ets-1. These changes were not evident when syndecan-2 was overexpressed. These findings suggest the involvement of IGF1R and Ets-1 in the induction of syndecan-2 synthesis and stimulation of proliferation by syndecan-1. This is the first report demonstrating that syndecan-1 enhances malignancy of a mesenchymal tumour cell line, via induction of syndecan-2 expression.

## Introduction

Syndecans are transmembrane proteoglycans bearing glycosaminoglycan (GAG) chains on their extracellular protein domain [1]. The ectodomain and its GAG chains have been reported to bind several extracellular matrix components [2] and other, cell surface proteins [3], [4], [5]. In this way, syndecans function as modulators of stromal function and mediators of cell adhesion, although the precise modes of action remain elusive [6], [7]. The biological activity of syndecans is further modulated by proteolytic shedding, whereby the ectodomain with its GAG chains is liberated and becomes a soluble effector. The truncated core protein remains embedded in the cell membrane, although its fate and cellular function mediated by the remnant core protein is unclear [8].

Syndecan-1 (CD138) expression is typical of epithelia [9], while syndecan-2 is confined to mesenchymal cells [10]. Although characteristic to the epithelium, syndecan-1 has also been detected in the condensing mesenchyme during tooth [11], [12], limb [13] and lung [10] morphogenesis, in the adherent stages of B lymphocyte maturation [14], or in confluent cultures of human foetal lung fibroblasts [15].

Dysregulation of syndecan expression has been reported during tumour formation and progression. In a number of neoplasms, expression patterns of syndecan-1 [2] and syndecan-2 [16] characteristically correlate with the tumour stage and grade. While an extensive number of studies investigated the role of syndecans in carcinomas, little is known about their function in tumours of mesenchymal origin.

A number of mesenchymal tumours, especially the ones that show epitheloid morphology, express syndecan-1 [17]. In malignant mesothelioma, a connective tissue tumour with partial epitheloid differentiation, syndecan-1 is rarely expressed, and its presence is associated with a more epitheloid phenotype and longer survival [18], [19], [20]. Similarly, cultured malignant mesothelioma cells produce less syndecan-1 than benign mesothelial cells, regardless of their phenotypic differentiation [21].

**Figure 1:**
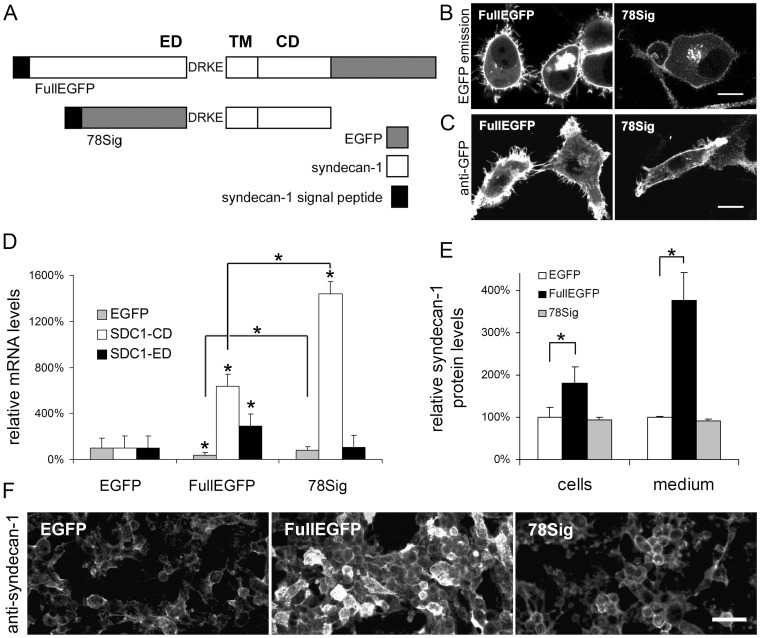
Detection of FullEGFP and 78Sig constructs and their effects on the expression and shedding of syndecan-1 in transfected HT-1080 cells. (A) FullEGFP represents the full-length syndecan-1/EGFP coding construct; 78Sig denotes the truncated syndecan-1 variant. ED, ectodomain; TM, transmembrane domain; CD, cytoplasmic domain. (B) Syndecan-1/EGFP fused proteins were detected by fluorescent confocal laser microscope on living HT-1080 cells 24 h after transfection. Scale bar: 10 µm. (C) Immunofluorescent staining of transfected HT-1080 cells by GFP specific monoclonal antibody on paraformaldehide-fixed and Triton X-100 permeabilised cells. Scale bar: 10 µm. (D) The mRNA expression of endogenous and recombinant syndecan-1 was examined by qRT-PCR with primer pairs specific for the cytoplasmic domain (SDC1-CD) and the ectodomain (SDC1-ED) or EGFP cDNA (EGFP). Latter one detects transcrips from all three plasmids thus provides information on transfection efficiency. In the course of relative quantification GAPDH served as reference gene, and the EGFP transfected sample as control. Results are expressed as mean±s.d. (n = 3), *p<0.05 versus control EGFP cells. Note, that the SDC-ED primers do not recognize the 78Sig cDNA, thus they detect only endogenous syndecan-1 in 78Sig transfectants. (E) Relative syndecan-1 protein levels in the lysates and in the media of cell cultures by CD138 ELISA that detects syndecan-1 with an ectodomain specific monoclonal antibody (n = 3), *p<0.05. (F) Immunofluorescent staining of transfected HT-1080 cells by syndecan-1 ectodomain specific monoclonal antibody B-B4 on methanol-fixed cells. Identical exposure times and background corrections were applied. Scale bar: 50 µm.

Overexpression of syndecan-2 enhances migration and invasion of HT-1080 cells into Matrigel [22]. In another type of mesenchymal tumour, osteosarcoma, syndecan-2 expression is reduced compared to that in osteoblasts and osteocytes in normal bone, and the expression of the proteoglycan sensitises tumour cells to basal and chemotherapy-induced apoptosis [23], [24].

Taken together, syndecans appear to be important players in oncogenesis therefore potential therapeutic targets in cancer [25]. Thus, identification of clinically relevant agonists or antagonists and understanding the molecular mechanisms underlying their action is of paramount importance. Earlier we reported that syndecan-1 increases proliferation and metastatic ability of HT-1080 cells [26]. In the current report, we expanded the scope of the studies by focusing on additional syndecan paralogues and their fine interplay.

**Table 1:** List of recognition sequences targeted by silencer plasmids used.

Plasmid name	Target sequence	NCBI Reference Sequence	Target position on ref. seq.
S1miRNA-a	CCGCAAATTGTGGCTACTAAT	NM_001006946.1	455–475
S1miRNA-b	ACCAAACAGGAGGAATTCTAT	NM_001006946.1	1298–1318
S2miRNA-a	GGGAGCTGATGAGGATGTAGA	NM_002998.3	789–809
S2miRNA-b	CGAAGAGGATACAAATGTGTA	NM_002998.3	990–1010
LacZ-miRNA	GACTACACAAATCAGCGATTT	–	–

S1miRNA-a and -b are specific for syndecan-1, S2miRNA-a and -b target syndecan-2.

## Materials and Methods

### Plasmid Constructs

For the FullEGFP plasmid construct (Szilák Labor Ltd., Szeged, Hungary), enhanced green fluorescent protein (EGFP) cDNA has been cloned in frame with the C-terminus of full length human syndecan-1. In case of the 78Sig plasmid construct (Szilák Labor Ltd.), a truncated syndecan-1 cDNA, lacking the ectodomain with the exception of the membrane proximal DRKE sequence, has been cloned in frame with the C-terminus of EGFP (Figure 1A). The host vector pEGFP-N1 (BD Biosciences, Clontech, Paolo Alto, CA, USA) was used as negative control for overexpression experiments. Plasmids coding for full length syndecans without an EGFP tag were also generated in-house (syndecan-2) or obtained (syndecan-1,Szilák Labor Ltd. and syndecan-4 [27]).

For gene silencing experiments, vector based RNA interference (RNAi) was applied using the BLOCK-iT™ Pol II miR RNAi system (Invitrogen by Life Technologies, Carlsbad, CA, USA). The inserts, containing the antisense target sequence (mature miRNA RNAi sequence), a miRNA loop derived from miR-155, and the sense target sequence with one base deletion were designed with the BLOCK-iT RNAi Designer (Invitrogen). The sense and antisense strands of the inserts were obtained as conventional oligonucleotides and annealed to double strands. Designed double strands were then cloned into the Block-iT™ pcDNA6.2-GW/EmGFP-miR Expression Vector (Invitrogen) according to the supplied protocol (BLOCK-iT™ Pol II miR RNAi Expression Vector Kit with EmGFP). The system allows fluorescent detection of successfully targeted cells. S1miRNA-a, -b and S2miRNA-a, -b plasmids encoded artificial microRNAs targeting human syndecan-1 or syndecan-2, respectively (Table 1). Two microRNA coding constructs were designed for each target. LacZmiRNA, supplied by the manufacturer, targeting β-D-galactosidase was used as a negative control for the silencing experiments.

### Cell Culture and Transfection

HT-1080 human fibrosarcoma cells (ATCC number: CCL-121) were grown in RPMI-1640 medium (Sigma-Aldrich, St Louis, MO, USA) containing 10% foetal bovine serum (FBS, from Sigma-Aldrich), and penicillin-streptomycin (100U and 100 µg/mL, respectively). Cells were cultured in 75 cm^2^ tissue culture flasks (Sarstedt, Newton, NC, USA) at 37°C under a humidified atmosphere containing 5% (v/v) CO_2_. Culture medium was changed twice per week, and cells were regularly tested for mycoplasma infection by PCR assay [28].

For transfection, 3×10^5^ HT-1080 cells were seeded into six-well plates and incubated for approximately 24 hours to reach 60–80% confluence. To achieve optimal transfection, 2 µL of FuGENE 6 transfection reagent (Roche, Basel, Switzerland) and 0.8 µg of plasmid DNA were diluted in 60 µL serum- and antibiotics-free RPMI-1640 medium. After 15 minutes of incubation, the mixture was added dropwise onto the cells preincubated in 1 mL of serum- and antibiotics-free RPMI. After 4 hours of incubation, the transfection mixture was removed from the cultures and replaced with normal culture medium.

To obtain stable transfectants, cells were selected by addition of Geneticin (G418, Roche) at a final concentration of 600 µg/mL, or in case of microRNA-coding plasmids, 20 µg/mL blasticidin (Invitrogen). Drug-resistant cells transfected with the BLOCK-iT vectors were sorted for EGFP-positive cells by fluorescence-activated cell sorting (FACS) using the BD FACSAria Flow Cytometer (BD Biosciences).

**Table 2:** List of primers used.

Name of primer pair	Primer sequences (5′-3′ orientation)	primer position on ref. seq.	NCBI Reference Sequence
EGFP	F: ATCGAGCTGAAGGGCATCG	367–385	gb|GU112756.1|
	R: CCTTGATGCCGTTCTTCTGCT	467–487	gb|GU112756.1|
SDC1-ED	F: GCTGACCTTCACACTCCCCA	908–927	NM_001006946.1
	R: CAAAGGTGAAGTCCTGCTCCC	1011–1031	NM_001006946.1
SDC1-CD	F: GAAGAAGAAGGACGAAGGCAG	1225–1245	NM_001006946.1
	R: CCTCCTGTTTGGTGGGC	1294–1310	NM_001006946.1
SDC2	F: AATGGACCCAGCCGAAGAG	978–996	NM_002998.3
	R: CAGCAATGACAGCTGCTAGGAC	1051–1072	NM_002998.3

F: forward primer, R: reverse primer.

### Subcellular Localisation of Recombinant EGFP Fusion Proteins

Distribution of syndecan-1/EGFP fusion proteins in living HT-1080 cells was examined 24–48 h after transfection using the MRC-1024 confocal laser scanning microscope (Bio-Rad, Hercules, CA, USA). The instrument was equipped with a Kr/Ar Laser and emission of signals was detected at 522±16 nm.

### Proliferation Assay

Stably transfected cells were seeded into 96-well plates at a density of 5000 cells/well. To quantify cells, the sulforhodamine B (SRB) colorimetric assay was used. This assay is based on the measurement of cellular protein content. Cells were fixed with 10% (w/v) trichloroacetic acid and stained with SRB (Sigma-Aldrich) for 30 minutes followed by repeated washing with 1% (v/v) acetic acid to remove the excess dye. The protein-bound dye was dissolved in a 10 mM Tris base solution and absorbance was determined at 570 nm using Multiskan MS ELISA plate reader (A.A. Lab Systems, Ramat-Gan, Israel). Doubling time of cells was calculated from the log phase of their growth curves measured by the SRB assay at 4, 24, 48 and 72 hours after seeding.

### Chemotaxis Assay

HT-1080 cell migration was measured using a 48-well micro chemotaxis chamber (Neuro Probe, Gaithersburg, MD, USA) equipped with a 8 µm pore-size polycarbonate membrane filter (Whatman, GE Healthcare Bio-Sciences Corp., Florham Park, NJ, USA). Fifty µL of cell suspension (1×10^6^ cells/mL) in culture medium containing 10% FBS was placed into the upper chamber. Extracellular matrix (ECM) gel from Engelbreth-Holm-Swarm murine sarcoma (Sigma-Aldrich) diluted in serum-free medium at 0.05 mg/mL was placed in the bottom chamber as chemoattractant, except for the blank sample where it was omitted. After a 4-hour-long incubation in humidified atmosphere and 5% CO_2_ at 37°C, the chamber was disassembled and cells from the upper face of the filter were dislodged. The membrane was dried, methanol-fixed and stained with Toluidine Blue (Sigma-Aldrich). The amount of cells migrated through the membrane was quantified by densitometry using the Kodak Image Station 4000MM (Carestream Health, Inc., Rochester, NY, USA) and software provided by the manufacturer.

### Animal Experiments and Lung Metastasis Model

All animal study protocols were conducted according to the Semmelweis University guidelines for animal care. Animal studies were approved by Budapest and Pest county Agricultural Administrative Authority Directorate for the Safety of the Food Chain and Animal Health, permit number 399/003/2005.

To generate spontaneous lung metastases, HT-1080 tumour cells were transplanted into the foot pads of mice [29]. HT-1080 cells stably transfected with EGFP, FullEGFP or 78Sig were injected into the right foot pads of five female severe combined immunodeficient (SCID) mice per group (10^5^ cells/mouse). Tumour volume was measured in two dimensions using a digital caliper and calculated according to Feldman J. [30] with the following formula: V  =  π/6×1.63 (length × width)^3/2^. For the FullEGFP injected group average tumour volume reached 150 mm^3^ at day 24, for the 78Sig group at day 29 and for the control EGFP group at day 34. To prevent toxic effect of tumour necrosis, tumour bearing legs were amputated when the average tumour size in the group reached the volume of 150 mm^3^. At the time of amputation mice were anesthetised with ketamine (80 mg/kg) combined with xylazine (12 mg/kg). All mice were sacrificed on day 50 after inoculation. The percentage of total lung area occupied by metastases was calculated by morphometrical analysis of hematoxylin-eosin (HE) stained sections of the lungs in 5 randomly chosen planes using the Kodak Image Station 4000MM and software provided by the manufacturer.

### RNA Isolation and Quantitative RT-PCR

Transfected cells stably overexpressing EGFP, FullEGFP or 78Sig were cultured for 48 hours, then total RNA was isolated using the RNeasy Mini Kit (Qiagen, Hilden, Germany), according to the protocol provided by the manufacturer. The yield and purity of the isolated RNA were estimated by an ND-1000 spectrophotometer (NanoDrop Technologies, Wilmington, DE, USA). The integrity and size distribution of the total RNA purified were analysed using Experion RNA Chips and the Experion Automated Electrophoresis Station (Bio-Rad).

To prove that HT-1080 cells were successfully transfected with syndecan-1 variants, quantitative reverse transcription PCR (qRT-PCR) was performed. Complementary DNAs (cDNAs) were generated from 1 µg of total RNA by M-MLV Reverse Transcriptase kit (Invitrogen) according to the instructions of the supplier. qRT-PCR was performed in the ABI Prism 7000 Sequence Detection System (Applied Biosystems by Life Technologies, Carlsbad, CA, USA), using primers at 1 µM final concentration, and Power SYBR® Green PCR Master Mix (Applied Biosystems by Life Technologies). The SDC1-CD primer pair was designed to amplify the cytoplasmic domain region of syndecan-1 cDNA, the SDC1-ED pair was specific for the ectodomain, while the SDC2 primers were specific for syndecan-2 (Table 2). TaqMan Gene Expression Assay for GAPDH was used as reference (Applied Biosystems by Life Technologies). All samples were run in triplicates in 20 µL reaction volume with 20 ng of cDNA/reaction. Results were obtained as threshold cycle (C_T_) values. Expression levels were calculated by using the 2^−ΔΔCT^ method.

### Oligoarray Analysis

Purified RNA samples were processed for oligoarray analysis using the Cancer PathwayFinder Oligo GEArray (SABiosciences, Qiagen, Hilden, Germany) representing 117 genes involved in cancer. Two-and-a-half µg of total RNA was labelled with the True-Labeling 2.0 Kit (SABiosciences), and hybridisation was performed according to the manufacturer’s protocol.

### CD138 ELISA and Western Blot

Total protein was extracted from cultures of HT-1080 transfectants and frozen primary tumour tissues. Primary tumours were homogenised in liquid nitrogen followed by addition of 1 mL of lysis buffer [20 mM Tris pH = 7.5, 2 mM EDTA, 150 mM NaCl, 1% Triton X-100, 2 mM sodium orthovanadate, 10 mM sodium fluoride, 0.5% protease inhibitor cocktail (Sigma-Aldrich)]. Cultured cells were washed with phosphate buffered saline (PBS) and lysed in 1 mL lysis buffer. Cells were scraped off and lysates were transferred into microcentrifuge tubes. After brief sonication and incubation on ice for 30 minutes, samples were centrifuged at 15,000 g for 20 minutes. The protein concentrations in the supernatants were measured according to Bradford [31]. Media of cultured cells were concentrated to ¼ of the original volume using the Amicon Ultra-15 Centrifugal Filter Unit with Ultracel-10 membrane (Millipore, Billerica, MA, USA).

The amounts of syndecan-1 in cultured cells, conditioned media, and tumour lysates were measured using a CD138 sandwich ELISA kit (Diaclone, Gen-Probe Incorporated, San Diego, CA, USA), according to the manufacturer's instructions. Samples were measured in triplicates.

For Western blotting, proteins were isolated as described above. In case of syndecan-2 Western blots, additional heparitinase digestion was performed as described before [32]. Twenty micrograms of total protein was mixed with loading buffer containing β-mercaptoethanol and incubated at 95°C for 5 minutes. Denatured samples were loaded onto 10% polyacrylamide gels and were run for 30 minutes at 200 V on Mini Protean vertical electrophoresis equipment (Bio-Rad). Proteins were transferred to PVDF membranes (Millipore) by blotting for 16 hours with 75 mA at 4°C. Ponceau staining was applied to determine the efficiency of protein transfer. Membranes were blocked with 3% (w/v) non-fat dry milk (Bio-Rad) in Tris-buffered saline (TBS) for 1 hour followed by incubation with primary antibodies (Table S1) at 4°C for 16 hours. GAPDH served as loading control. Membranes were washed 5 times with TBS containing 0.5 v/v% Tween-20, then were incubated with appropriate HRP-conjugated secondary antibodies (Dako, Glostrup, Denmark) at room temperature for 1 hour. Signals were visualised and documented by the Kodak Image Station 4000 MM (Carestream Health, Inc.).

### Phospho-receptor Tyrosine Kinase Array

Stably transfected HT-1080 cells were screened for 42 different phosphorylated receptor tyrosine kinases (RTKs) using the Proteome Profiler™ antibody array (R&D Systems, Catalog Number ARY001) following the manufacturer’s instructions. Positive signals were captured and quantified by Kodak Image Station 4000 MM.

### Immunofluorescent Staining

For immunofluorescent staining, 3×10^5^ transfected cells were seeded onto glass coverslips in 6-well plates and cultured for 24–48 hours, then fixed in methanol. Cryostat sections of the primary tumours were also fixed in ice-cold methanol. Samples were then washed in PBS, blocked with 5% (w/v) bovine serum albumin (BSA) at 37°C for 30 minutes. After washing, samples were incubated with the appropriate primary antibody (Table S1) diluted in PBS, containing 1% (w/v) BSA at 37°C for 1.5 hours. Appropriate fluorescent secondary antibodies were applied at room temperature for 30 minutes. Nuclei were stained with 4′,6-diamidino-2-phenylindole (DAPI) or propidium iodide. Pictures were taken by a Nikon Eclipse E600 microscope and Lucia Cytogenetics version 1.5.6 software, or by an MRC-1024 confocal laser scanning microscope. Equal exposure times and background corrections were applied for all images.

### Flow Cytometry

Cells were harvested with 5 mM EDTA (in PBS), and fixed in ice cold ethanol for 5 minutes. Samples were then washed in PBS, blocked with 3% (w/v) BSA in PBS at 37°C for 30 minutes. To detect syndecans, cells were directly incubated with an Alexa Fluor® 647-conjugated specific antibody against syndecan-1 (clone B-B4) or a non-conjugated syndecan-2 antibody (M-140, and ZMD.308)(Table S1). ZMD.308 binding was visualised using a Cy5 conjugated anti-rabbit antibody from Jackson ImmunoResearch Laboratories Inc. (West Grove, PA, USA). Stained cells were loaded onto a FACScalibur flow cytometer (BD Biosciences) and quantitated with Cell Quest software (BD Biosciences). A range of 5000-10,000 events were measured from all samples.

### Statistical Analysis

Differences between means were evaluated using two-tailed unpaired Student’s *t*-test after F-probe, or Mann-Whitney U-test. χ^2^-test was applied for differences between various groups of in vivo experiments. Statistical significance was considered at p<0.05.

**Figure 2:**
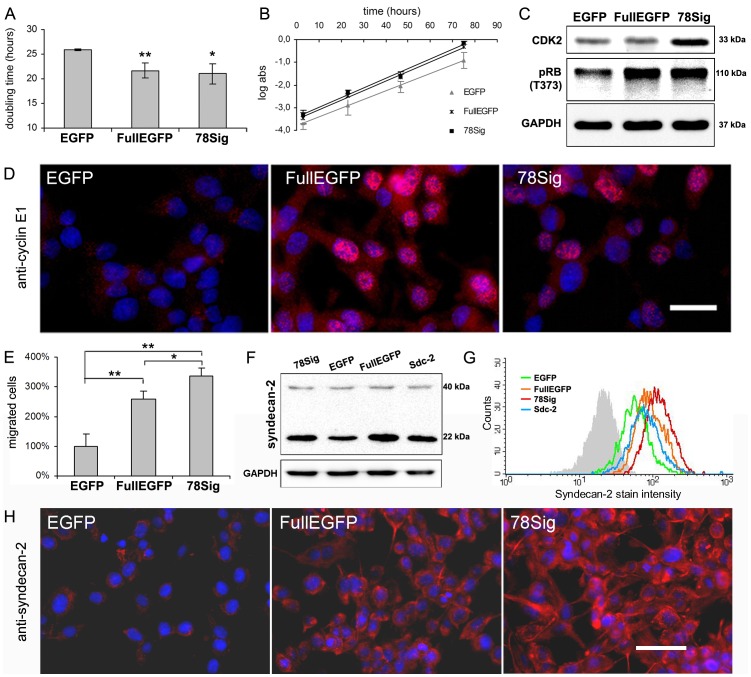
Effects of FullEGFP and 78Sig on the proliferation and chemotactic migration of HT-1080 cells. (A) Doubling times were calculated from the log phase of growth curves (B) obtained from the SRB colorimetric assay. Values are expressed as mean±s.d. (n = 8). (C) CDK2, phospho-retinoblastoma (at T373 position) and GAPDH immunoblots of HT-1080 cells transfected with EGFP, FullEGFP or 78Sig. (D) Representative fields of cyclin E immunocytochemistry (red) on cultured HT-1080 cells transfected with EGFP, FullEGFP or 78Sig. Scale bar: 20 µm. (E) Relative amounts of migrated cells toward ECM proteins in a Boyden chamber after transfection with EGFP, FullEGFP or 78Sig. Values are shown as mean±s.d. (n = 5), *p<0.05 and **p<0.01. (F) Syndecan-2 and GAPDH immunoblots of cultured HT-1080 transfectants. Sdc-2 stands for syndecan-2 transfection. (G) Results of flow cytometry after immunofluorescent staining of stable transfectants using the ZMD.308 antibody for syndecan-2. (H) Immunofluorescent staining of methanol-fixed cells by the syndecan-2 specific antibody L-18 (red). Identical exposure times and background corrections were applied. Scale bar: 50 µm. For (E and G) nuclei were counterstained with DAPI (blue).

## Results

### EGFP-tagged Syndecan-1 Variants Express and Localise to the Cell Membrane

Syndecan-1/EGFP plasmid vectors (Figure 1A) were stably transfected into HT-1080 cells.

Recombinant chimeric syndecan-1/EGFP proteins were detected in living cells by their green fluorescence, using confocal laser microscopy 24 hours after transfection. Control cells transfected with empty EGFP vector exhibited diffuse cytoplasmic and nuclear fluorescence (not shown), whereas FullEGFP and 78Sig proteins localised to the cell membrane and the endomembrane system (Figure 1B). The green fluorescence of 78Sig cells was less intensive than that of FullEGFP. Anti-GFP immunofluorescent staining displayed similarly strong membrane signals (Figure 1C).

### FullEGFP Increases the Amount of Cell Surface-bound and Soluble Syndecan-1, whereas 78Sig does not Affect Expression or Shedding of Endogenous Syndecan-1

Results obtained from qRT-PCR using primers specific for EGFP revealed that there were a significantly higher number of transcripts of 78Sig, than those of FullEGFP in HT-1080 transfectants (Figure 1D). Accordingly, FullEGFP construct induced a 6-fold, and 78Sig a 14-fold increase in total syndecan-1 mRNA levels, when compared to the control EGFP, detected by the SDC1-CD primer pair that is specific for the syndecan-1 cytoplasmic domain. The SDC1-ED primer pair amplifies only the full length syndecan-1 cDNA whereas it is unable to recognise the 78Sig transcript. This primer pair detected increased mRNA levels only in the FullEGFP transfectants, indicating that the 78Sig construct did not influence the expression of endogenous syndecan-1 (Figure 1D).

In each microgram of total protein isolated from EGFP, FullEGFP, and 78Sig transfectants and their conditioned cell media 315±13, 570±8, 294±8 and 5.5±0.02, 20.5±0.72, 5.0±0.06 nanograms of syndecan-1 could be detected, respectively, using CD138 ELISA specific for the ectodomain. When data were normalised to the EGFP controls, twice as much syndecan-1 protein was found in the cell lysate and four times more in the medium of FullEGFP transfectants, while no changes in 78Sig transfectants have been detected (Figure 1E). These results supported the data obtained by qRT-PCR.

Immunofluorescent staining on stable transfectants was performed using the B-B4 antibody specific for the ectodomain of syndecan-1 (Figure 1F). In comparison with the EGFP transfected cells, only FullEGFP cells showed stronger staining, fully supporting the ELISA and qRT-PCR results.

**Figure 3:**
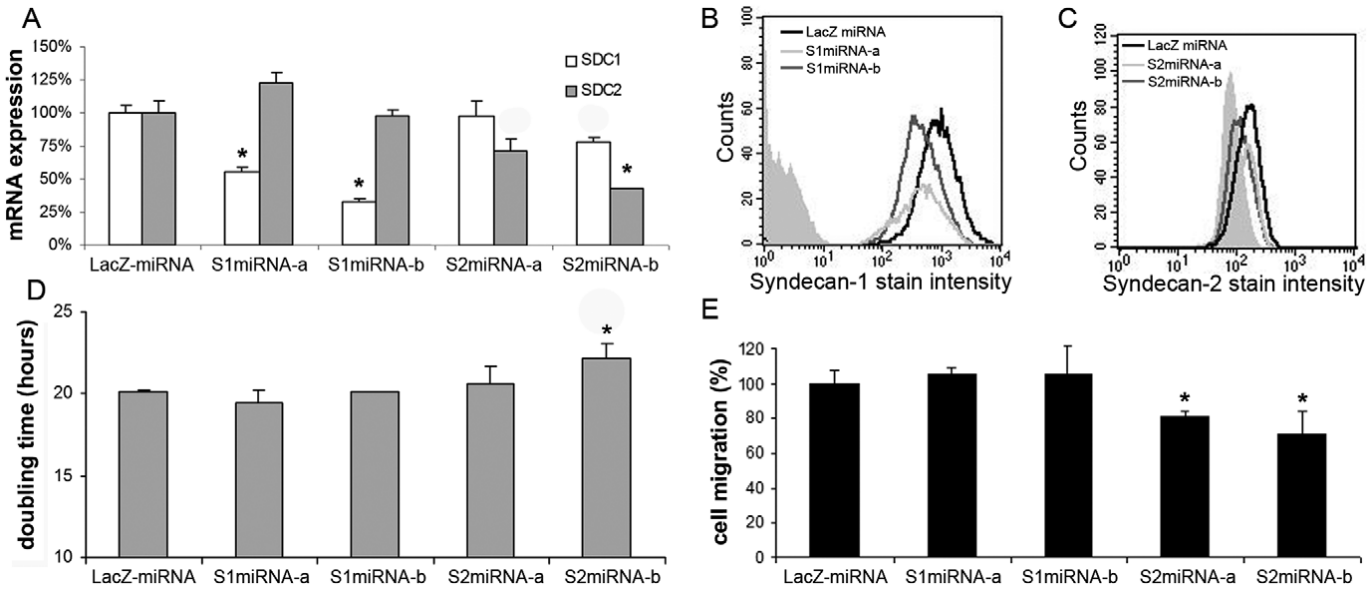
Effects of syndecan-1 and -2 gene silencing on the proliferation and migration of HT-1080 cells. (A) Relative mRNA levels of syndecan-1 (SDC1) and syndecan-2 (SDC2) in HT-1080 cells transfected with artificial microRNA coding plasmids, specific for β-D-galactosidase (LacZ), syndecan-1 (S1miRNA-a and -b) or syndecan-2 (S2miRNA-a and -b). Values are expressed as mean±s.d calculated by relative quantification of three independent qRT-PCR results using GAPDH as reference gene and LacZmiRNA control as calibrator. (B, C) Results of flow cytometry after immunofluorescent staining of stable transfectants using the B-B4 antibody for syndecan-1 or the M-140 antibody for syndecan-2. (D) Doubling times of stable transfectants. Values are expressed as mean±s.d. (n = 8). (E) Relative amounts of migrated HT-1080 stable transfectants in a 48-well Boyden chamber. The chemoattractants were ECM proteins. Values are expressed as mean±s.d. (n = 5), *p<0.05 versus control LacZmiRNA cells.

**Figure 4:**
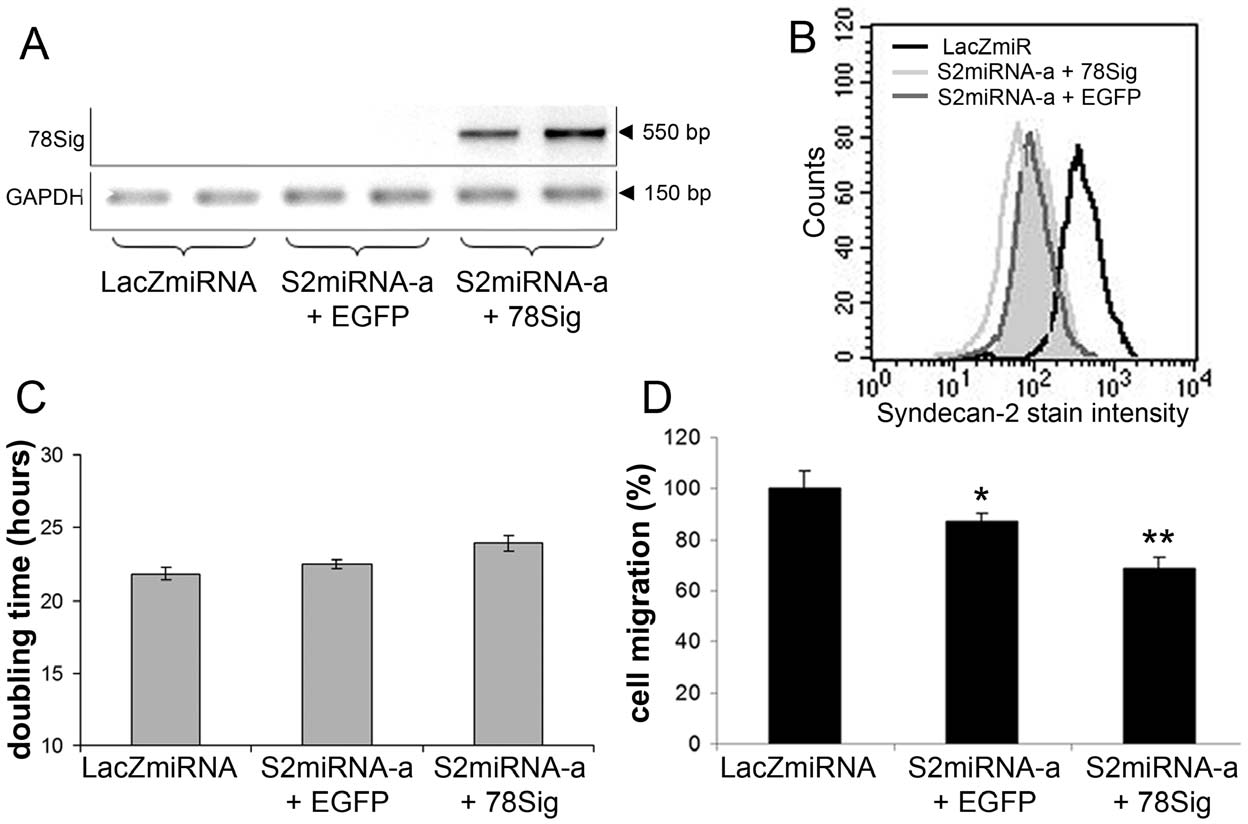
Truncated syndecan-1 does not affect the proliferation or the migration of HT-1080 cells if syndecan-2 is silenced. (A) Detection of transcripts of the 78Sig construct in double transfectants after Geneticin selection by RT-PCR using the EGFP forward and the SDC1-CD reverse primers that produce a 550 bp long amplicon. Agarose electroforetograms the PCR products of these primers. GAPDH was used as positive control. S2miRNA-a + EGFP: the S2miRNA-a was co-transfected with the EGFP control vector. S2miRNA-a +78Sig: S2miRNA-a was co-transfected with the 78Sig vector. (B) Cell sorting results after immunofluorescent staining of double transfectants using the M-140 antibody specific for syndecan-2. (C) Doubling times of double transfectants. Values are expressed as mean±s.d. (n = 8). (D) Relative amounts of migrated HT-1080 stable transfectants in 48-well Boyden chamber. Chemoattractants were ECM proteins. Values are expressed as mean±s.d. *p<0.05 and **p<0.05 versus LacZmiRNA cells (n = 5).

### FullEGFP and 78Sig Both Increase Proliferation and Chemotactic Migration –Involvement of Cyclin E1/CDK2 Complex and Syndecan-2

Proliferative activity of transfectants was measured by the SRB assay, and doubling times (Figure 2A) were calculated from the log phase of proliferation curves (Figure 2B). Both syndecan-1 constructs stimulated proliferation of HT-1080 cells. While for control EGFP the doubling time was 25.6±0.2 h, it was reduced to 21.7±1.5 by FullEGFP and to 21.0±2.0 h by 78Sig and these differences were statistically significant (p<0.05 and p<0.01, Figure 2A).

Oligonucleotide arrays were performed in order to characterise signalling activities transmitting the effects of syndecan-1 in HT-1080 cells. Primarily, genes that regulate the G1/S transition of the cell cycle showed altered mRNA expression, including elevated CDK2 and cyclin E1 mRNA levels (data not shown). The increase of CDK2 expression was verified by Western blotting (Figure 2C), and cyclin E1 expression by immunofluorescent staining (Figure 2D). CDK2 when in complex with cyclin E1 phosphorylates retinoblastoma 1 (Rb). Thus, levels of Rb phosphorylated at Thr373 were evaluated by immunoblotting. Phosphorylation of Rb protein was enhanced in both FullEGFP and 78Sig cells relative to the EGFP control (Figure 2C).

To study the effects of full length and truncated syndecan-1 on the migration ability of HT-1080 cells, the Boyden chemotaxis assay was performed. Directed migration of HT-1080 cells transfected with FullEGFP and 78Sig towards ECM proteins was significantly higher than that of EGFP control used as baseline values (p<0.01). The 78Sig enhanced migration the most intensively, significantly more than FullEGFP did (p<0.05) (Figure 2E). In addition, using the ZMD.308 antibody an increase in syndecan-2 protein expression was found in cells overexpressing full length or truncated syndecan-1, detected by Western blotting (Figure 2F) and also by FACS analysis. Fluorescent signals of the antibody were increased by 1.7 fold by FullEGFP and by 2.4 fold by the 78Sig (Figure 2G). These upregulations of syndecan-2 were also detected by immunofluorescent staining where L-18 antibody was used to mark syndecan-2 (Figure 2H).

**Figure 5:**
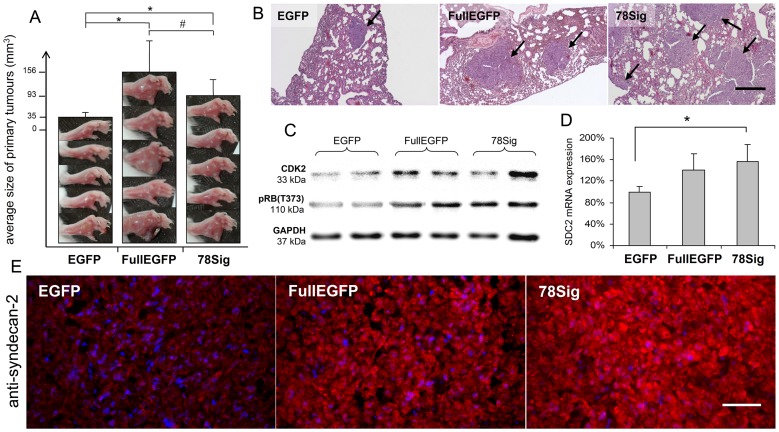
Effects of FullEGFP and 78Sig on the malignancy of HT-1080 cells in vivo. (A) Size of primary tumours in the footpads of SCID mice on the 24^th^ day after injection of HT-1080 cells expressing EGFP, FullEGFP or 78Sig. Photographs and results of morphometric analysis are shown. *p<0.05 (*t*-test), #p<0.05 (χ^2^-test)(n = 5). (B) Histological appearance of lung metastases (arrows) of HT-1080 transfectants. Images do not represent the area percentage of metastases; rather, the only one small EGFP tumour found is shown. HE-stained sections are shown, scale bar: 200 µm. (C) CDK2, phospho-retinoblastoma (at T373 position) and GAPDH immunoblots from HT-1080 primary tumours stably expressing EGFP, FullEGFP or 78Sig. (D) Relative syndecan-2 mRNA levels of the transfectants. Values are expressed as mean±s.d calculated by relative quantification of three independent qRT-PCR results using GAPDH as reference gene and EGFP transfected control as calibrator. *p<0.05 (non-parametric Mann-Whitney test) compared to EGFP control cells. (E) Immunofluorescent staining of methanol-fixed frozen sections by the syndecan-2 specific antibody L-18. Identical exposure times and background corrections were applied Scale bar: 50 µm.

**Table 3:** Size of primary tumours on the 24th day after inoculation of stably transfected HT-1080 cells (mm^3^).

Mouse No.	EGFP	FullEGFP	78Sig
1	38.9	154.4	81.1
2	32.6	74.9	68.3
3	28.4	241.5	83.0
4	22.2	66.7	61.2
5	52.9	243.9	170.1

HT-1080 cells transfected with the corresponding plasmid construct were injected into the foot pads of 5 mice. The volume of primary tumours was measured for all mice.

**Table 4:** The area percentage of lung metastases.

		area percentage of lung metastases				
group	mouse No.	plane 1	plane 2	plane 3	plane 4	plane 5
EGFP	1	0,00	0,00	0,00	0,00	0,00
	2	0,00	0,00	0,00	0,00	0,00
	3	0,00	0,00	0,00	0,00	0,00
	4	0,00	0,00	0,00	0,00	0,00
	5	0,46	0,00	0,00	0,00	0,00
FullEGFP	1	0,00	0,00	0,00	0,00	0,00
	2	35,34	12,53	20,08	14,90	21,48
	3	58,69	0,00	56,52	0,00	50,45
	4	19,55	3,78	3,08	4,64	12,55
	5	0,00	0,00	0,12	1,87	0,00
78Sig	1	65,29	41,30	53,31	48,92	56,39
	2	0,00	0,00	0,00	0,00	0,00
	3	58,45	10,37	37,41	17,76	41,25
	4	0,00	0,00	0,00	0,00	0,00
	5	0,91	0,00	0,00	0,00	0,00

Mice were sacrificed on the 50th day after injection of HT-1080 cells into their foot pads expressing EGFP, FullEGFP or 78Sig. Area percentage referring to the area fractions of lung metastases were calculated by morphometrical analysis of hematoxylin-eosin stained sections of the lungs in 5 random planes.

### Syndecan-1 Enhances Migration and Proliferation in Cooperation with Syndecan-2

Overexpression of full length or truncated syndecan-1 resulted in subsequent increase in syndecan-2 expression (Figure 2F, 2G, 2H). As overexpression of syndecan-2 has been shown to significantly increase chemotaxis of HT-1080 cells [22], in the current report we tested a functional relationship between syndecan-1 and syndecan-2.

To refine the hierarchy of syndecans in this context, vector based RNA interference assays using artificial microRNA coding plasmids specific for syndecan-1 and syndecan-2 (S1miRNA-a and -b, and S2miRNA-a and -b) were utilised. LacZmiRNA plasmid served as control (Table 1). S1miRNA-a and -b resulted in a significant decrease in syndecan-1 expression to 56% and 33%, respectively (p<0.05), while no significant changes in the expression of syndecan-2 was detected. S2miRNA-a and -b reduced syndecan-2 expression to 72% and 43%, respectively. Silencing effects were evidenced by qRT-PCR using the SDC1-ED and SDC2 primer pairs and GAPDH for reference gene (Figure 3A). Syndecan-1 protein levels also decreased by S1miRNA-a and -b, measured by FACS using direct labelled B-B4 antibody (Figure 3B). Reduction in syndecan-2 expression by S2miRNA-a and -b was noted using the syndecan-2 specific M-140 antibody (Figure 3C). Only S2miRNA-b affected doubling times increasing it significantly (Figure 3D). Syndecan-2 silencing significantly inhibited chemotaxis of HT-1080, whereas syndecan-1 knock-down had no such effect (Figure 3E).

**Figure 6:**
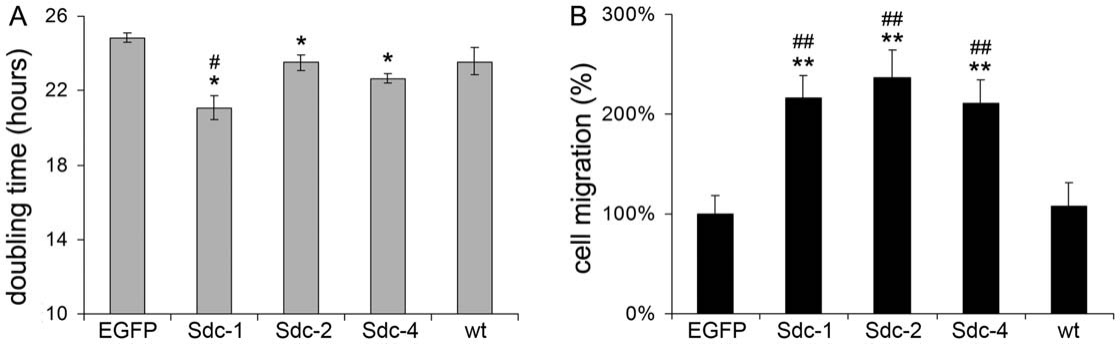
Effects of syndecan-1, -2 and -4 overexpression on the proliferation and migration of HT-1080 cells. (A) Doubling times of stable transfectants were calculated from the log phase of growth curves obtained from SRB colorimetric assays. Values are expressed as mean±s.d. (n = 8). (B) Relative amounts of migrated cells toward ECM proteins in a Boyden chamber. Values are shown as mean±s.d. (n = 5). (A-B) EGFP denotes cells transfected with the empty pEGFP-N1 vector. Sdc-1, Sdc-2 and Sdc-4 refer to syndecan-1, -2 and -4 with no EGFP-tag, respectively, and wt to untransfected wild type (wt) cells. Symbols are *p<0.05 versus EGFP, **p<0.01 versus EGFP, #p<0.05 versus wt, and ##p<0.01 realtive to wt.

**Figure 7:**
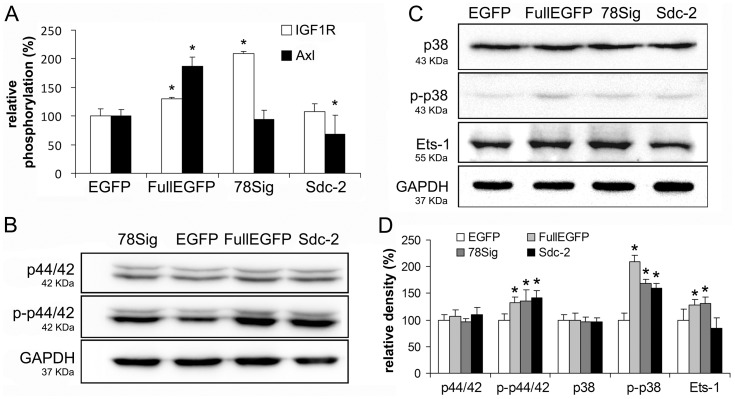
Candidates in the molecular mechanism of the cooperation between syndecan-1 and syndecan-2. (A) Results of pRTK array. Relative extents of IGF1R and Axl phosphorylation in HT-1080 transfectants. Values are expressed as mean±s.d. *p<0.05 versus control EGFP cells (n = 3). (B and C) Representative immunoblots from HT-1080 cells stably expressing EGFP, FullEGFP, 78Sig or Sdc-2. Antibodies used are listed in Table S1. (D) Results of densitometry of western blots. Values are expressed as mean±s.d. *p<0.05 by *t*-test.

To test the importance of syndecan-2 in the highly malignant phenotype of syndecan-1 overexpressing fibrosarcomas, S2miRNA-a was co-transfected with 78Sig or an EGFP empty vector control. The presence of 78Sig transcripts in “S2miRNA-a +78Sig” stable transfectants was confirmed by RT-PCR, using the EGFP forward and the SDC1-CD reverse primers, while “S2miRNA-a + EGFP” and LacZmiRNA control cells were negative for the transcript. For positive control, GAPDH primers were used (Figure 4A). The expression of syndecan-2 was successfully reduced in both “S2miRNA-a +78Sig” and “S2miRNA-a + EGFP” cells, compared to LacZmiRNA control cells, as evidenced by FACS using the M-140 antibody (Figure 4B). When co-transfected with the S2miRNA-a construct, 78Sig increased neither the proliferation rate nor the chemotaxic ability of the cells (Figure 4C, D). Chemotaxis was inhibited in both “S2miRNA-a + EGFP” and “S2miRNA-a +78Sig” cells, compared to that in LacZmiRNA cells (Figure 4D).

### FullEGFP and 78Sig Promotes Malignancy of HT-1080 Cells in Vivo

To estimate in vivo malignancy of the stable transfectants, they were injected into foot pads of SCID mice (5 per group). Twenty-four days after injection, the average volume of primary tumours increased as follows: EGFP (35.0±11.7 mm^3^), FullEGFP (156.3±86.0 mm^3^), and 78Sig (92.8±44.2 mm^3^) (Figure 5A). After 1 day, significant differences between the EGFP control and the syndecan-1 transfectant groups were found in the size of primary tumours by *t*-test (*p<0.05). Moreover, in the FullEGFP group, a significantly higher number of primary tumours reached the size of 150 mm^3^ than those in the 78Sig group, according to the χ^2^-test (#p<0.05) indicating that the FullEGFP construct promoted proliferation more effectively (Table 3). While the EGFP transfected group required 34 days to reach 150 mm^3^ average tumour volume, for 78Sig and FullEGFP it was only 29 and 24 days, respectively. Fifty days after injection, mice were sacrificed and the area fraction of lung metastases (Table 4) was calculated by morphometric analysis of 5 different, random planes of HE-stained sections (Figure 5B). In the EGFP group only one animal developed metastasis in its lung with negligible size. This number was 4 and 3 for FullEGFP and 78Sig groups, respectively (Table 4). The area of lung metastases in animals having tumours in the lung was 19.92±16.60% and 28.76±22.97% for FullEGFP and 78Sig, respectively, significantly more than the 0.02±0.15% of the control EGFP (p<0.05).

Immunofluorescent staining, Western blot and qRT-PCR were performed to explore whether molecular changes underlying the increased in vitro cell proliferation rate were detectable in primary tumours as well. Western blot assays (Figure 5C) confirmed increased CDK2 expression and enhanced retinoblastoma phosphorylation at T373. Furthermore, elevated transcription and protein expression of syndecan-2 in FullEGFP and 78Sig expressing tumours was confirmed by qRT-PCR (Figure 5D) and immunofluorescent staining (Figure 5E).

In order to confirm the presence of recombinant proteins in vivo, qRT-PCR assay with EGFP primers, and immunofluorescent staining for GFP were performed on primary tumours. Results of qRT-PCR relative quantification suggested that both syndecan-1/EGFP fusion constructs were transcribed (Figure S1A). Transfection with FullEGFP resulted in a 6-fold, and with 78Sig a 20-fold increase in syndecan-1 mRNA levels, compared to the control EGFP transfectant, using the SDC1-CD primer pair. The SDC1-ED primer pair detected increased mRNA levels in FullEGFP transfectants only (Figure S1B). To detect the recombinant proteins in primary tumours, immunofluorescent staining for GFP was performed (Figure S1C top row). FullEGFP expressing primary tumours exhibited stronger signals than those expressing EGFP or 78Sig, visualised by immunostaining with the B-B4 antibody specific for the syndecan-1 ectodomain (Figure S1C bottom row). These data confirmed the results found in cell cultures.

### Syndecan-1, -2, and -4 Stimulates Chemotaxis but only Syndecan-1 Enhances Proliferation of HT-1080 Cells

To expand the scope of our study, (and exclude possible interferences caused by the GFP-tag), HT-1080 strains stably expressing full length syndecan-1, -2, and -4 without a GFP-tag were established and their behaviour was tested in vitro. Syndecan-1 significantly reduced doubling time from 24.84±0,26 hours to 21.07±0,65 hours, compared with EGFP control cells. This effect was also significant when compared to untransfected wild type (wt) cells (p<0.05). Overexpression of syndecan-2 and -4 caused significant reduction in the doubling time only in comparison to EGFP cells (p<0.05), but not wt (Figure 6A).

Overexpression of either syndecan paralogue uniformly increased the migratory ability of HT-1080 cells up to two fold (p<0.01). Migration of EGFP control cells did not differ significantly from that detected using wt cells (Figure 6B).

### Implication for Insulin-like Growth Factor 1 Receptor (IGF1R) and Ets-1 Transcription Factor in the Cooperation of Syndecan-1 and Syndecan-2

To identify key signalling events underlying the observed induction of syndecan-2 by syndecan-1 overexpression in HT-1080, EGFP, FullEGFP, 78Sig and Sdc-2 stable transfectants were assayed on a phospho-receptor tyrosine kinase (pRTK) array and by Western blot.

According to these initial experiments two of the 42 RTKs investigated, IGF1R and Axl, showed considerable changes in their phosphorylation levels. When compared to the EGFP control cells, both receptors were significantly hyperphosphorylated in FullEGFP expressing cells, while only IGF1R showed an enhancement in 78Sig and Axl a moderate but significant reduction in the Sdc-2 transfectants (Figure 7A). MAP kinases p44-42 and p38 were hyperphosphorylated in FullEGFP, 78Sig as well as in Sdc-2 cells (Figures 7B and 7C). There were no changes in the phosphorylation of AKT (data not presented) while the expression of Ets-1 transcription factor was increased only in FullEGFP and 78Sig cells (Figure 7C and 7D).

## Discussion

Syndecan-1 is expressed principally in epithelial tissues; hence studies aiming to address its role in malignancies examine mostly carcinomas. Syndecan-1 modulates the biological behaviour of parenchymal cells in several types of carcinomas. Additionally, its stromal appearance has been identified as a prognostic factor [33], [34], [35], [36], [37], [38]. Recently the number of studies focusing on syndecan-1 in tumours of mesenchymal origin rapidly increases.

In the present study, we demonstrated that overexpression of either full-length or truncated syndecan-1 lacking the ectodomain enhances proliferation, migration and spontaneous metastasis formation ability using the human fibrosarcoma cell line HT-1080. Although we provide evidence that syndecan-1, syndecan-2 and -4 uniformly stimulate chemotactic migration of HT-1080 cells, according to our studies only syndecan-1 promotes proliferation when overexpressed.

We confirmed that both full length and truncated syndecan-1/EGFP fusion proteins appear on the cell surface. Although the green fluorescence of 78Sig is less intensive than that of FullEGFP, the truncated variant is expressed in a significantly higher level. The two constructs enhanced chemotaxis in different extents reflecting their different expression levels. It is important to note, that FullEGFP exert the same effects as the untagged full length syndecan-1, suggesting that the EGFP-tag on the cytoplasmic domain does not interfere with the cellular processes we investigated. Thus, the increasingly significant effect of truncated syndecan-1 on syndecan-2 expression and on cell migration is explained by its higher level of expression.

The measurement of ectodomain levels revealed that overexpression of 78Sig influenced neither expression nor shedding of endogenous syndecan-1. Overexpression of FullEGFP resulted in an increased amount of syndecan-1 fragments on the cell surface and consequently in the medium. Thus, putatively, transfection with either construct may result in the accumulation of syndecan-1 lacking the ectodomain. According to Endo [39], the cleavage of syndecan-1 ectodomain enhances migration of HT-1080 cells. Others described that a mutant syndecan-1, unable to shed, abolishes the pro-migratory role of syndecan-1 [40]. In support to these findings, we showed that syndecan-1 enhances cell migration in HT-1080 independently of the presence of its ectodomain.

An interesting cross-talk between syndecan-1 and -2 was found in the migration of HT-1080 cell line. According to Park [22], overexpression of syndecan-2 stimulates migration of these cells. We confirmed that transfection of syndecan-2 increase chemotactic migration of HT-1080 cells. Moreover, in our case migration toward ECM proteins was also stimulated by syndecan-1 transfection and additionally, syndecan-2 was simultaneously upregulated. Chemotaxis of HT-1080 was suppressed after syndecan-2 silencing, while downregulation of syndecan-1 had no such effect. The 78Sig construct was not able to exert its migration stimulating effect when co-transfected with a syndecan-2 silencer construct. This result proves, that syndecan-2 is a key player in the pro-migratory effect of syndecan-1. That is, syndecan-2 should be situated downstream of syndecan-1 in the motility signal cascade. Similar cooperation was found by silencing techniques between syndecan-2 and -4 in actin stress fibre formation in lung cancer cell lines P29 and LM66-H11 [41], or between syndecan-1 and syndecan-4 in the migration of dendritic cells after their phospholipid induced maturation [42]. Interestingly, overexpression but not silencing of syndecan-1 affected syndecan-2 expression and consequently migration of HT-1080. Syndecan-2 appears to have a basic threshold level that cannot be reduced by syndecan-1 silencing. The reason to this phenomenon may be that the linkage between syndecan-1 and -2 is indirect. We hypothesize that the presence of mediators acting between the two molecules transmit the effect of syndecan-1 on syndecan-2 expression. This presumption is supported by the observation that syndecan-1 increases syndecan-2 expression at transcription level as well (Figure 5D). Nevertheless, the promoter of syndecan-2 is certainly regulated also by other factors, not only by syndecan-1. In addition, others found in STAV-AB mesothelioma cells that transfection of syndecan-1– contrary to the case in HT-1080– downregulates syndecan-2 expression [43].

It is more difficult to identify the relation of syndecan-1 and -2 in the context of cell proliferation than that in migration. The reason of this may due to the less intensive effect of syndecan-1 overexpression on cell proliferation than that on cell migration. Overexpression experiments revealed that only syndecan-1 stimulates proliferation significantly in itself, syndecan-2 does not, suggesting that syndecan-1 has a distinct proliferation enhancing effect independent of syndecan-2. Nevertheless, silencing of syndecan-2 slightly inhibited the proliferation, and was able to inhibit the proliferative effect of the 78Sig construct, suggesting the prominent role of syndecan-2 in these processes. Apparently simultaneous overexpression of syndecan-1 and -2 is needed to stimulate proliferation of HT-1080 cells effectively.

In addition to their identical effects, both FullEGFP and 78Sig affected expression or phosphorylation of mostly the same genes involved in cell cycle regulation or migration suggesting similar mechanisms of action. In the background of increased cell proliferation after FullEGFP or 78Sig transfection, we detected upregulation of cyclin E1 as well as increased phosphorylation of Rb protein. Phosphorylation of Thr373 in Rb can be accomplished by the CDK2 cyclin E1 complex, and this phosphorylation alone is sufficient to inactivate the Rb protein [44]. Our findings suggest that transfection of syndecan-1 causes hyperphosphorylation of Rb by the CDK2 - cyclin E1 complex, an event that contributes to the accelerated proliferation of HT-1080. Treatment of HT-1080 cells with PD 98059 MAPK inhibitor leads to inhibition of the CDK2 - cyclin E1 complex and arrests the cell cycle in G1 phase [45] indicating the prominent role of this complex in HT-1080 cell proliferation.

Interestingly, in STAV-AB malignant mesothelioma and B6FS fibrosarcoma cells overexpression of the same full length and truncated syndecan-1 constructs exhibit opposite effects, causing reduced cell proliferation by the elongation of G1 and S phases of the cell cycle [43] and retarded migration [46]. These studies together with our work show that syndecan-1 can either accelerate or inhibit motility and cell cycle of mesenchymal cells even in the absence of the ectodomain, reflecting the dual role of syndecan-1 in mesenchymal tumours. This property of the three cell lines analyzed herein renders them a useful model in the future to unfold complex nature of syndecan-1 in malignancies.

To better understand the regulation of syndecan-2 expression by syndecan-1, alterations in expression or phosphorylation levels of various signal molecules were studied., These events occur only upon syndecan-1 overexpression and not when syndecan-2 is overexpressed. The receptor tyrosine kinase IGF1R and the transcription factor Ets-1 matched this criterion. Syndecan-1 has been demonstrated to interact with and activate IGF1R [47]. Ets-1 is a potential transcription factor to IGF1R [48], and it is involved in tumour invasion and metastasis [49] processes also regulated by syndecan-2 in HT-1080 cells. Surprisingly phosphorylation of p44/42 and p38 was stimulated by both syndecan-1 and -2 although proliferation was stimulated only by syndecan-1. This suggests that syndecan-1 could stimulate proliferation through an alternative mechanism that is MAP kinase independent. We have to stress that these preliminary results have to be confirmed by alternative experimental design protocols.

The significant increase in the motility of FullEGFP and 78Sig transfectants found in vitro by chemotaxis assays is in good accordance with their elevated in vivo metastatic potential. We found an increase in the number of mice developing lung metastasis and in the area fraction of metastases both in the FullEGFP and 78Sig groups.

In addition to the numerous similarities in the behaviour of full length and truncated syndecan-1 transfectants, some differences were also observed. Although the presence or absence of the ectodomain did not influence the proliferation enhancing effect of syndecan-1 in cell culture, in our mouse model the FullEGFP transfected tumours grew faster than the 78Sig expressors. The increased in vivo proliferation of these transfectants appears to be independent of syndecan-2 expression as FullEGFP cells contained less syndecan-2 than those expressing 78Sig. This suggests an additional, yet inidentified mechanism of full length syndecan-1 regulating HT-1080 proliferation in the presence of the ectodomain. Potentially this process is elicited by the tissue microenvironment.

Taken together, this work is the first to present syndecan-1 playing a tumour promoter role in a mesenchymal tumour cell line, a process that progreses through the cooperation of syndecan-1 and syndecan-2.

## Supporting Information

Figure S1 **Detection of EGFP and syndecan-1 in the primary tumours.** (A) mRNA expression of EGFP by qRT-PCR normalised to GAPDH levels by relative quantification. Results are expressed as mean±s.e.m. relative to EGFP control (n = 2). (B) mRNA expression of syndecan-1 was examined by qRT-PCR with primer pairs specific for the cytoplasmic domain (SDC1-CD) and the ectodomain (SDC1-ED) the control was the EGFP in the course of relative quantification. Results are expressed as mean±s.d. (n = 3), *p<0.05 versus control EGFP cells. (C) Immunostaining on frozen sections with anti-GFP antibody (top row), or with the B-B4 antibody specific for syndecan-1 (bottom row). Images were captured by confocal laser microscopy and were handled equally applying same background correction. Scale bar: 50 µm.
(TIF)

Table S1 **Primary antibodies applied.**
(DOC)
