# Supplementary figures and images for: Syndecan-1 Enhances Proliferation, Migration and Metastasis of HT-1080 Cells in Cooperation with Syndecan-2
Source: PLoS One. 2012 Jun 26;7(6):e39474. doi: 10.1371/journal.pone.0039474 (PMC3383727; doi:10.1371/journal.pone.0039474)

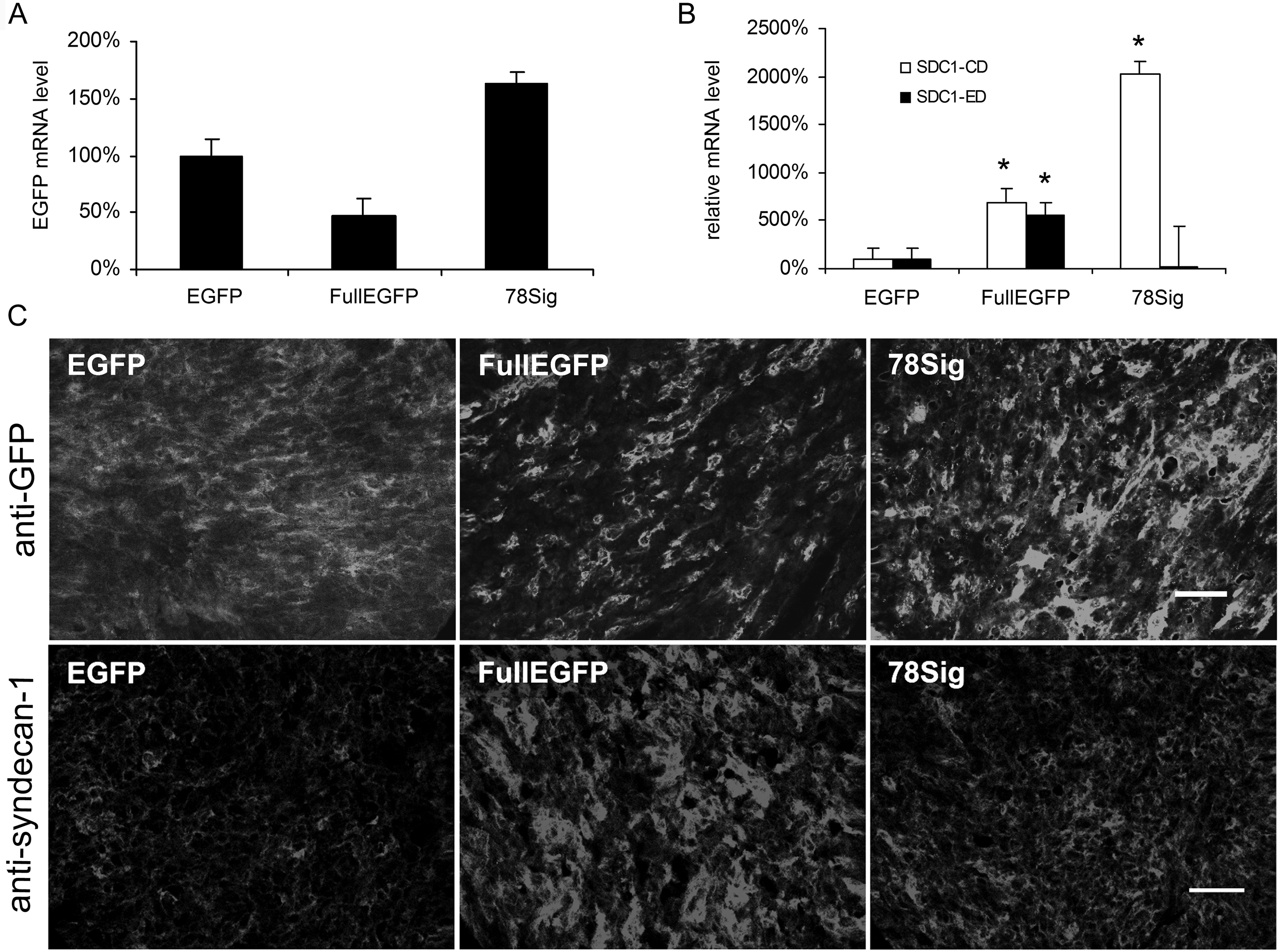

Supplement: Figure S1 — Detection of EGFP and syndecan-1 in the primary tumours. (A) mRNA expression of EGFP by qRT-PCR normalised to GAPDH levels by relative quantification. Results are expressed as mean±s.e.m. relative to EGFP control (n = 2). (B) mRNA expression of syndecan-1 was examined by qRT-PCR with primer pairs specific for the cytoplasmic domain (SDC1-CD) and the ectodomain (SDC1-ED) the control was the EGFP in the course of relative quantification. Results are expressed as mean±s.d. (n = 3), *p<0.05 versus control EGFP cells. (C) Immunostaining on frozen sections with anti-GFP antibody (top row), or with the B-B4 antibody specific for syndecan-1 (bottom row). Images were captured by confocal laser microscopy and were handled equally applying same background correction. Scale bar: 50 µm. (TIF) [file pone.0039474.s001.tif]
