# Supplementary material for: Syndecan-1 Enhances Proliferation, Migration and Metastasis of HT-1080 Cells in Cooperation with Syndecan-2
Source: PLoS One. 2012 Jun 26;7(6):e39474. doi: 10.1371/journal.pone.0039474 (PMC3383727; doi:10.1371/journal.pone.0039474)
Supplement: Table S1 — Primary antibodies applied. (DOC) [file pone.0039474.s002.doc]

**Table S1. Primary antibodies applied**

| Antigen | Host species, isotype | Antibody ID No. | Clone | Application | Cat. No. | Company | Dilution |
| --- | --- | --- | --- | --- | --- | --- | --- |
| GFP | mouse, monoclonal IgG1κ | - | 7.1; 13.1 | IF | 1 814 460 | Roche | 1:200 |
| syndecan-1 | mouse, monoclonal | - | B-B4 | IF | MCA681 | AbD Serotec | 1:100 |
| syndecan-1 | mouse, monoclonal | - | B-B4 | FC | MCA681A647 | AbD Serotec | 1:10 |
| syndecan-2 | goat, polyclonal IgG | L-18 | - | WB IF | sc-9492 | Santa Cruz | 1:500, 1:100 |
| syndecan-2 | rabbit, polyclonal IgG | ZMD.308 | - | WB | 36-6200 | Invitrogen | 1:500 |
| syndecan-2 | rabbit, polyclonal IgG | M-140 | - | FC | sc-15348 | Santa Cruz | 1:50, 1:100 |
| cyclin-E1 | mouse, monoclonal IgG2a | Ab-5 | 13A3 | IF | MS-1060 | Lab Vision | 1:100 |
| CDK2 | rabbit, polyclonal IgG | - | - | WB | C5223 | Sigma | 1:1000 |
| pRb(T373) | rabbit, monclonal IgG | - | EP821Y | WB | ab52975 | Abcam | 1:5000 |
| GAPDH | mouse, monoclonal IgG1 | - | FF26A/F9 | WB | MCA2427 | AbD Serotec | 1:2000 |
| p44/42 MAPK | rabbit, polyclonal | - | - | WB | 9102 | Cell Signaling Technology | 1:500 |
| pp44/42 MAPK (T202/Y204) | rabbit, monoclonal | D13.14.4E | - | WB | 4370 | Cell Signaling Technology | 1:500 |
| p38 MAPK | rabbit, polyclonal |  |  | WB | 9102 | Cell Signaling Technology | 1:100 |
| pp38 MAPK | rabbit, monoclonal |  |  | WB | 4370 | Cell Signaling Technology | 1:100 |
| Ets-1 | rabbit, polyclonal | H-20 |  | WB | Sc-350 | Santa Cruz | 1:250 |

WB: Western blotting, IF: immunofluorescence, FC: flow cytometry
